# Whole‐organ transdermal photobiomodulation (PBM) of COVID‐19: A 50‐patient case study

**DOI:** 10.1002/jbio.202100194

**Published:** 2021-11-22

**Authors:** Richard K. Williams, John Raimondo, David Cahn, Aldon Williams, Daniel Schell

**Affiliations:** ^1^ Applied BioPhotonics Ltd. Cupertino California USA; ^2^ Pulmonair San Antonio Texas USA; ^3^ O'Connor Hospital San Jose California USA; ^4^ Advanced Spinal Pain Management Boerne Texas USA; ^5^ LightMD, Inc. Cupertino California USA

**Keywords:** ARDS, COVID‐19, dyspnea, mitochondria, photobiomodulation, red/NIR LED, SpO_2_, transdermal

## Abstract

A nonrandomized 50‐person case study of COVID‐19‐positive patients was conducted employing (for the first time) a regimen of whole‐organ deep‐tissue transdermal dynamic photobiomodulation (PBM) as a primary (or exclusive) therapeutic modality in the treatment of coronavirus. Therapy sessions comprised algorithmically alternating red (650 nm) and near‐infrared (NIR; 850 nm) LEDs with an average irradiance of 11 mW/cm^2^ dynamically sequenced at multiple pulse frequencies. Delivered via 3D bendable polymeric pads maintaining orthogonal optical incidence to body contours over 1,000 cm^2^, a single 84‐minute session concurrently delivered 20 kJ to the sinuses and 15 kJ to each lung at skin temperatures below 42°C. Therapeutic outcomes observed include significant reductions in the duration and severity of disease symptoms. Acute conditions including fever, body aches (BA) and respiratory distress comprising paroxysmal coughing; lung congestion, dyspnea and hypoxia; sinus congestion; acute eye inflammation; and extreme malaise were eliminated in 41/50 patients within 4 days of commencing PBM treatments with 50/50 patients fully recovering within 3 weeks with no supplemental oxygen requirements. SpO_2_ concentrations improved as much as 9 points (average 2.5 points) across the entire study population. The PBM sessions required to completely resolve COVID‐19 conditions appears monotonically correlated to the time‐to‐treatment (TTTx)—the delay between the onset of a patient's symptoms and commencing PBM therapy. In contrast, acute inflammatory symptoms were resolved within 4 days irrespective of TTTx.
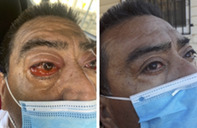

## Graphical abstract

In a 2020 nonrandomized outpatient case study, 50 symptomatic COVID‐19‐positive patients received noninvasive deep‐tissue whole‐organ PBM therapy comprising 84‐minute concurrent anterior treatments of lungs and sinuses using red and NIR LEDs pulsed in multifrequency algorithms. After two sessions, therapeutic outcomes showed acute symptomatic relief of paroxysmal coughing; lung congestion dyspnea; and hypoxia; and sinus inflammation in 82% of the study irrespective of initial severity and 3‐week full recovery for all patients.

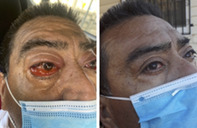

## 1 INTRODUCTION

Photobiomodulation (PBM) represents a new and emerging therapeutic modality for the treatment of coronavirus[1], [2] (including COVID‐19) and related inflammatory diseases such as pneumonia,[3], [4], [5] acute respiratory distress syndrome (ARDS),[6], [7], [8] chronic obstructive pulmonary disease (COPD)[9] and acute lung injury[10]. In PBM therapy, diseased or injured tissue is illuminated by a source of nonionizing electromagnetic radiation or EMR (delivered via lasers or LEDs) to stimulate physiologically beneficial photochemical reactions. Although small amounts of heat are produced during PBM, the process is photochemical not thermal, invoking biophysical mechanisms offering therapeutic outcomes that thermotherapy cannot.[11] Reported PBM benefits include reduced tissue inflammation[12], [13], [14] and dyspnea, [15] improved circulation,[16] decreased pain[17] and accelerated recovery.[18], [19]

PBM is also found to interfere with a pathogen's ability to replicate[20] through innate immune response (via reactive ion species, [ROS]) and over longer intervals by regulating adaptive immune response. Other light wavelengths can damage pathogens directly,[21], [22], [23] albeit not transdermally in deep tissue or viscera.

PBM can be used adjunctively with other remedies, as a primary therapeutic modality, in palliative care[24], [25] and for prophylaxis[26], [27]. In the treatment of COVID‐19, the primary goal of PBM is symptomatic relief—to restore breathing; reduce (or break) fever; ameliorate coughing; mitigate aches and pains; shorten the duration of the viral infection; combat pneumonia or other secondary infections; reduce mucus, edema and congestion in the bronchia, alveoli and sinuses; and circumvent the need for ventilation.[18] Compared to pharmacological regimens, PBM is not harmful to kidneys, liver or stomach and is nonaddictive. Unlike antibiotics and antivirals, pathogens cannot develop immunity to photon energy, the “medicine” of PBM. Unfortunately, the therapeutic administration of PBM by physicians or clinicians today remains problematic, primarily because of fundamental design deficiencies in commercially available PBM apparatus, including both lasers and LED systems.

### 1.1 Obstacles to whole‐organ PBM

Without scanning, the characteristically small spot size of a laser is incapable of concurrent whole‐organ PBM treatment, requiring a nurse or specially trained therapist to constantly hold and manually reposition a handheld probe or wand repeatedly atop the patient dozens of times in order to cover a large treatment area. Invariably, this manual operation results in poor energy (dose) uniformity[28], [29] across the lungs, liver or other large organs. Aside from being uncomfortably slow, the arduous process of manual probing necessitates a nurse spending entire shifts working in extremely close proximity (less than 0.5 m) to severely ill patients and increasing cross infection risks for therapists and patients alike.[30], [31] Moreover, treating the face, sinuses and upper respiratory tract with laser light unavoidably presents the risk of eye damage or blindness to the patient. Lasers, especially at high power, also pose possible eye injury risks from reflected light to the nurse administering the treatment.[32], [33], [34] Scanning lasers[35] suffer from high costs, complex apparatus, fragile optics, and low throughput.

LED‐based PBM, in contrast, overcomes the small spot size and safety risk issues associated with lasers, beneficially allowing a treatment to proceed unsupervised after setup. Unfortunately, the optical efficiency of photon delivery in most LED apparatus limits their efficacy, particularly in deep tissue therapy. Specifically, rigid LED panels (along with light beds and light walls) are incapable of significant energy coupling into deep tissue, rendering them unsuitable in the treatment of organs or disease. Unable to maintain a perpendicular angle of incidence along body contours, the majority of optical energy emitted from LED panels is off‐axis, lost to reflection, refraction and scattering in the epithelial layer, never penetrating into the visceral organs where therapy is required.

Studies report a gap of only 2.5 cm between a rigid panel and treated skin loses 94% of the penetrating energy of the impinging photons.[36] As such, rigid light sources are fundamentally unsuitable for deep tissue and large organ therapy. Commercial LED systems also lack the capability of ensuring uniform LED brightness (poor dose control), dynamically controlling pulse modulation rates (for enhancing tissue specificity), sequencing multiple wavelength LEDs (to control penetration depth), or adjusting the array's optical power output (to regulate skin temperature). Together, these PBM parameters represent key factors in controlling PBM total dose—the energy distribution of photons in treated tissue. With these important photonic conditions left uncontrolled, treatments cannot be expected to produce consistent therapeutic outcomes. Furthermore, most LED PBM apparatus do not qualify as medical‐grade products as they lack integral fail‐safe safety systems; have not earned proper medical, good manufacturing practice (GMP) and Federal Communications Commission/electromagnetic compatibility (FCC/EMC) certifications; and employ porous materials unsuitable for aseptic medical use or regular disinfection.

To our knowledge, this case study is the first to employ a medical‐grade LED PBM apparatus designed specifically to overcome the energy‐coupling and power control deficiencies of existing systems. As such, the study is the first to evaluate concurrent whole‐organ PBM using large‐area deep‐tissue LED PBM in the treatment of disease. Specifically, its goal is to determine whether the use of whole‐organ dynamic PBM is capable of the efficacious treatment of COVID‐19 and its symptoms.

### 1.2 PBM mechanisms of action

PBM is the biophysical mechanism whereby light of specific wavelengths interacts with biomolecules in living cells and cellular organelles to invoke a photochemical reaction with physiological consequences, that is, photons modulating cellular metabolism. To reach internal organs, the photons must first traverse the body's outer tissue without being absorbed by bodily fluids, water or blood. Penetrating through the skin into the body cavity, photons impinging on an organ must be absorbed by a specific class of light‐sensitive molecules to affect physiological function. These photon‐absorbing molecules, called chromophores,[37], [16] generally comprise transmembrane proteins, ion pumps and ion gates located on the surfaces of, or within, cells and cellular organelles (including mitochondria). Chromophores are biologically ubiquitous, contained in nearly every living cell and tissue type in animals including neurons, muscles, epithelial, connecting tissue, and vascular systems.

Not all absorbed photons are, however, capable of invoking PBM. Photons carry discrete amounts of energy called quanta, having a magnitude proportional to EMR frequency and inversely proportional to wavelength. Governed by quantum mechanics, absorbed photons must possess a minimum amount of energy to make and break biochemical bonds[38] in order to induce PBM. As a rule of thumb, the threshold of PBM corresponds to an energy greater than the energy carried by adenosine triphosphate (ATP), around 0.6 eV, corresponding to light having wavelengths shorter than 2,000 nm just beyond the near‐infrared (NIR) spectrum. So, while visible and NIR light can invoke PBM, longer wave infrared behaves as heat and does not meet the minimum requirement for PBM.[11]

Just because a photon carries sufficient energy to stimulate PBM does not mean it can reach an internal organ. Photon transport and absorption within tissue depend on light wavelength. In general, longer wavelength EMR (eg, infrared and red light) penetrates to greater tissue depths than shorter wavelengths (such as blue, violet or ultraviolet). Contrary to classical Newtonian physics, longer wavelength EMR carries less energy but penetrates more deeply than shorter wavelength higher‐energy photons. For photons to reach and then be absorbed by internal organs, tissue must be sufficiently transparent at a given wavelength to allow light to penetrate beyond subdermal layers but be sufficiently opaque to be absorbed by chromophores in the targeted tissue.

If a photon is too long in wavelength (and therefore too low in energy) to invoke PBM, the absorbed light acts like a heat lamp raising the tissue temperature but not stimulating photochemical reactions.[39] At shorter wavelengths, however, photons absorbed by chromophores cause PBM, eliciting electrical, ionic and chemical transformations within the cell and releasing waste heat (molecular kinetics) as a by‐product. The magnitude of self‐heating, however, is minimal. At optical power densities considered safe, that is, for steady‐state irradiances below approximately 10 to 15 mW/cm^2^ of average power, skin temperature is naturally regulated by heart rate, blood perfusion, sweating, and convective cooling to a comfortable 42°C. Heat, however, is not the mechanism of action for PBM.

As a photochemical process, PBM involves reactions requiring energy one to two orders of magnitude greater than the thermal energy available during thermotherapy. As such, thermotherapy cannot produce the same therapeutic outcomes as PBM. References to PBM as “therapeutic heating” or describing PBM apparatus as heat lamps is scientifically erroneous and phenomenologically misleading. During PBM, waste heat and far‐infrared blackbody radiation may assist in catalyzing reactions thermodynamically to improve chemical kinetics but only as a by‐product of PBM. In other words, some degree of thermotherapy occurs locally in treated tissue during PBM.

Given the foregoing penetration and energy requirements, deep‐tissue PBM beneficial for treating visceral organs is limited to a narrow spectrum of wavelengths in the red and NIR portion of the EMR spectrum. Located between the optical absorption wavelengths for water and for deoxygenated hemoglobin, this so‐called transparent optical window in animals spans the range from 650 nm to 950 nm. Curiously, the same wavelengths correspond to the reported action spectra of cytochrome c oxidase (CCO), a light‐absorbing chromophore representing the fourth functional group in the electron transport chain of the mitochondrial membrane protein cytochrome c. Functionally, CCO is responsible for regulating NO, controlling the generation of ATP, maintaining cellular metabolism, and homeostasis and controlling gene expression through nuclear transcription factors (NTFs).

During PBM, light changes the mitochondrial membrane potential (MMP) inducing increased production of ATP and elevating the local concentrations of tissue ROS. Reactive oxide species such as superoxide (O^*−*^), NO, and H_2_O_2_ play pivotal roles in innate immune response irrespective of an invading contagion. Light also affects mast cells and, through degranulation, induces angiogenesis. The role of PBM in stem cell generation and differentiation is only now emerging. Despite this myriad of photochemical pathways, it is primarily through mitochondria that PBM modulates cellular metabolism and respiration.

Although mitochondria are believed to be morphologically identical throughout all tissue types, their abundance in various organs and their influence on physiology is tissue specific. For example, in motor and cardiac function, mitochondria manage the kinetics of muscle contraction; in signal transduction, they control neurons and nociceptors; and in tissue, circulatory and lymph systems, they help manage both innate and adaptive immune response. By sensing divalent calcium gradients, mitochondria regulate necrotic (and apoptotic) cell death and replacement (fibroblastic remodeling) in wound repair, neurogenesis, angiogenesis and bone growth. Ion gate conduction is frequency dependent, involving reaction rates spanning the audio spectrum.

For example, although electron transport and neurological communication react in milliseconds, ion transport across membranes involved in vasodilation inflammatory response innate immune response; and homeostatic regulation occur 10 to 100 times slower. By controlling the dynamic modulation rate of PBM to match specific physiological mechanisms and tissue types, and adjusting the treatment regimen accordingly, PBM therapies can be selectively tuned to maximize immune response; suppress inflammatory processes; improve blood perfusion; control cellular necrosis, apoptosis and phagocytosis; and to stimulate granulation and structural remodeling through fibroblastic repair, thereby expediting the entire inflammatory process of healing. It is for this reason that continuous‐wave (CW) lasers generally produce subpar results compared to pulse‐modulated PBM delivery and why no one modulation frequency is best for all organs and tissues. It also explains why PBM response is biphasic, where too much energy delivered too quickly can inhibit rather than promote healing, particularly for high‐fluence CW laser operation. Modulation rate's effect on biphasic response is unknown.

Healing and immune response are even slower. For example, ATP synthesis peaks 6 hours after PBM, while nuclear transcription and protein synthesis involved in tissue repair and adaptive immune response can take 36 to 48 hours following PBM. PBM therapeutic regimens synchronized to physiological response times, for example, with sessions administered every 2 or 3 days, have been found to be more efficacious than more aggressive treatment schedules.

## 2 EXPERIMENTAL DESIGN

### 2.1 Study strategy

Given the complex epidemiology of the COVID‐19 pandemic and rapidly evolving SARS‐CoV‐2 genomic variants, over the past year, it has become painfully obvious no one‐size‐fits‐all therapeutic stratagem can effectively counter the contagion's infectivity, address its symptomatic diversity, or contain its spread throughout global populations. While some countries are experiencing declines in infection rates (particularly those deploying aggressive vaccination programs), other countries are in the midst of a fourth or even fifth surge.

As such, the intent of this case study is to develop a pragmatic approach to address acute medical conditions associated with the SARS CoV‐2 pathogen and its physiological impact, focused on the most dangerous manifestations of the disease, generally those involving severe inflammation, cytokine storms, respiratory distress, low blood oxygen and the propensity for thrombosis arising from increased blood viscosity. A secondary goal of the study was to determine whether the duration of severity and infectivity could be reduced to more quickly free up hospital beds, essentially increasing hospital capacity without incurring the cost or delays of new infrastructure.

Under medical standards of care and compassionate use, therapies known to produce beneficial therapeutic outcomes cannot ethically be withheld from patients seeking urgent relief. In the midst of a pandemic outbreak, therefore, the incoming patients should not be denied treatment just to maximize a target test population or to select a specific set of conditions. Accordingly, patients were treated without regard to meeting defined study criteria, irrespective of comorbidities. Instead, the population of treated patients was analyzed in accordance with well‐established symptomatic criteria of COVID‐19 comprising three broad and nonexclusive categories of predominant conditions, namely (i) pulmonary distress, (ii) upper respiratory infection and (iii) other symptoms.

Patients experiencing pulmonary distress include acute bronchitis, severe coughing, shortness of breath, low SpO_2_ levels, pneumonia and/or COPD or ARDS‐like symptoms, chest pain, and increased lung opacity in chest X‐rays. Upper respiratory distress includes severe sinus congestion and rhinitis; sore throat; choking; excessive mucus; burning, itching and inflamed eyes; blurry vision; severe sinus congestion; and persistent headache (HA). The category “other symptoms” primarily comprise gastrointestinal distress including nausea, vomiting, diarrhea, stomach cramping, painful urination, kidney pain or persistent abdominal discomfort (AbD). Fever, restless sleep, and malaise were not indicative as these symptoms were common to all groups treated.

### 2.2 PBM apparatus

In this study, whole‐organ dynamic PBM therapy was performed using the ABPT1003 phototherapy system specified, designed and manufactured by Applied BioPhotonics Ltd., comprising a software‐based dual‐output controller driving up to six 3D bendable silicone pads containing dense arrays of LEDs. The PBM controller comprises medically rated galvanically isolated offline power supplies, redundant safety systems, graphical user interface/user experience with color LCD touchscreen and hierarchical menu and a microprocessor‐controlled LED drive system for dynamically sequencing LED wavelengths and modulating frequencies in accordance with defined algorithms. In operation, modulating pulse frequency, optical power density and skin temperature are actively controlled using pulse width modulation with nanosecond precision in accordance with predefined treatment or session protocols.

**FIGURE 1:**
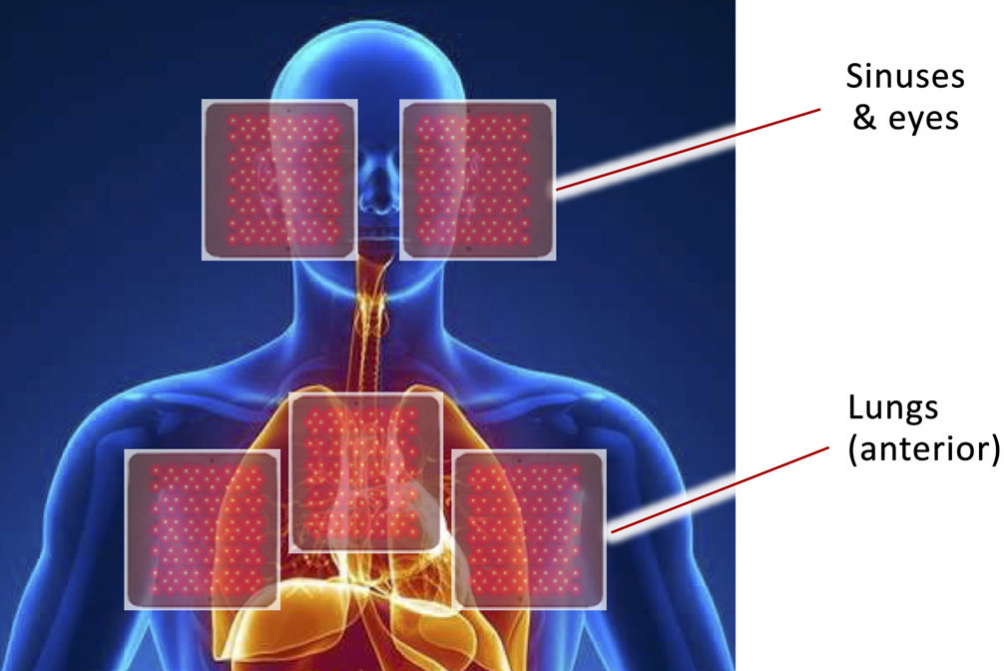
Schematic representation of anterior positioning of LED pads atop sinuses and lungs (attribution: Encyclopedia Britannica [human body])

Optical energy is delivered by 3D bendable LED pads containing arrays of red and NIR LEDs having wavelengths of 650 nm and 850 nm, respectively, within the aforementioned transparent optical window corresponding to reported action spectra of CCO. During conduction, LED current is maintained at constant levels independent of stochastic variations in LED forward voltages or component aging, facilitating precise brightness consistency and uniformity within a pad, from pad‐to‐pad, and from one manufacturing batch to another. During therapy, skin temperature maintains 42°C in equilibrium, safely delivering the maximum power density in steady‐state operation. The optical power level may also be adjusted for cooler tissue temperatures as desired for patient comfort. Collectively, the system's six lightpads contain an array of over 1,200 LEDs covering up to a total conformal surface area of more than 1,200 cm^2^. The large pad treatment area facilitates concurrent treatment of whole organs (such as lungs and sinuses shown in Figure 1) without the need for scanning or for a therapist to manually hold or reposition the light source during therapy.

The specially designed LED pads bend in three dimensions, conforming to body contours to maintain orthogonal optical incidence, preventing energy loss or reduction in penetration depth due to reflection, refraction and surface scattering. Except for a transparent plastic or silicone sanitary barrier, the LED pads fit snugly against the body with virtually no gap. The device is cleared by the US Food and Drug Administration (FDA), Taiwan FDA, Dubai Health Authority and UAE Ministry of Health and certified for manufacturing made in accordance with US and Taiwan GMP standards. The manufacturer passed a 1‐week random audit by the US FDA including an exhaustive review of the product's Design History File, Device Master Record, total quality management systems and on‐site factory audits. The device is FDA Global Unique Device Identification Database registered. Independent safety certifications include Conformitè Europëenne, International Organization for Standardization‐13485, International Electrotechnical Commission‐62471 photobiological safety and FCC approval for EMC. The photobiomodulation therapy system (including controller, LED pads and dynamic algorithms) is covered by an intellectual property portfolio comprising 21 issued patents via the United States Patent and Trademark Office, European Patent Office and other international authorities. Another dozen patent remain pending.

### 2.3 PBM therapeutic regimen

Considering COVID shelter‐in‐place provisions during the 2020 outbreak, patients in the study were treated on an outpatient basis in well‐ventilated areas rather than in hospitals and indoor clinics, thereby eliminating risks of patient‐to‐patient cross infection. To minimize direct contact with the administering physician, patients maintained a physical separation from the doctor as facilitated by 2.5‐m‐long electrically shielded cables connecting the LED pads to the PBM controller. No doctors or therapists became inflected during this study.

During pretreatment setup, patients were instructed to position two sets of pads on their anterior, one set‐of‐three pads covering their lungs and the second set‐of‐two (or three) pads placed across the face, eyes and sinuses as shown previously in Figure 1. All pads were driven concurrently using identical treatment algorithms comprising fully automated dynamic sequencing of red and NIR LEDs modulated at predefined pulse patterns developed to address specific physiological conditions. Each session involved a series of treatments comprising the following protocol intended to:

1. Stimulate immune response locally and systemically (24 minutes)
2. Increase local circulation in treated organs and tissue (20 minutes)
3. Promote homeostasis in treated organs and tissue (20 minutes)
4. Relieving inflammation in treated organs and tissue (20 minutes)

Requiring 84 minutes to complete, at 11 mW/cm^2^, each session delivers 50 J/cm^2^. For patient comfort, Steps 3 and 4 can be shortened by 10 minutes each as required with minimal impact on therapeutic efficacy. The specified therapeutic regimen involves performing one session every 2 or 3 days until the patient experiences significant relief.

### 2.4 Study population

In this study, 67 patients were recommended for treatment on referral basis. Of the incoming patient population, 50 symptomatic individuals tested positive for COVID while another 9 individuals were symptomatic but unconfirmed for coronavirus infection (either being untested or initially receiving a negative COVID test outcome). Another eight untested patients were asymptomatic but had been in direct contact with symptomatic COVID‐positive patients and requested to be treated prophylactically. In accordance with medical standards of care, all 67 patients were (as requested) accepted for and treated with PBM therapy. Only the 50 incoming patients testing COVID‐positive, however, were considered as members of the nonrandomized case study.

**FIGURE 2:**
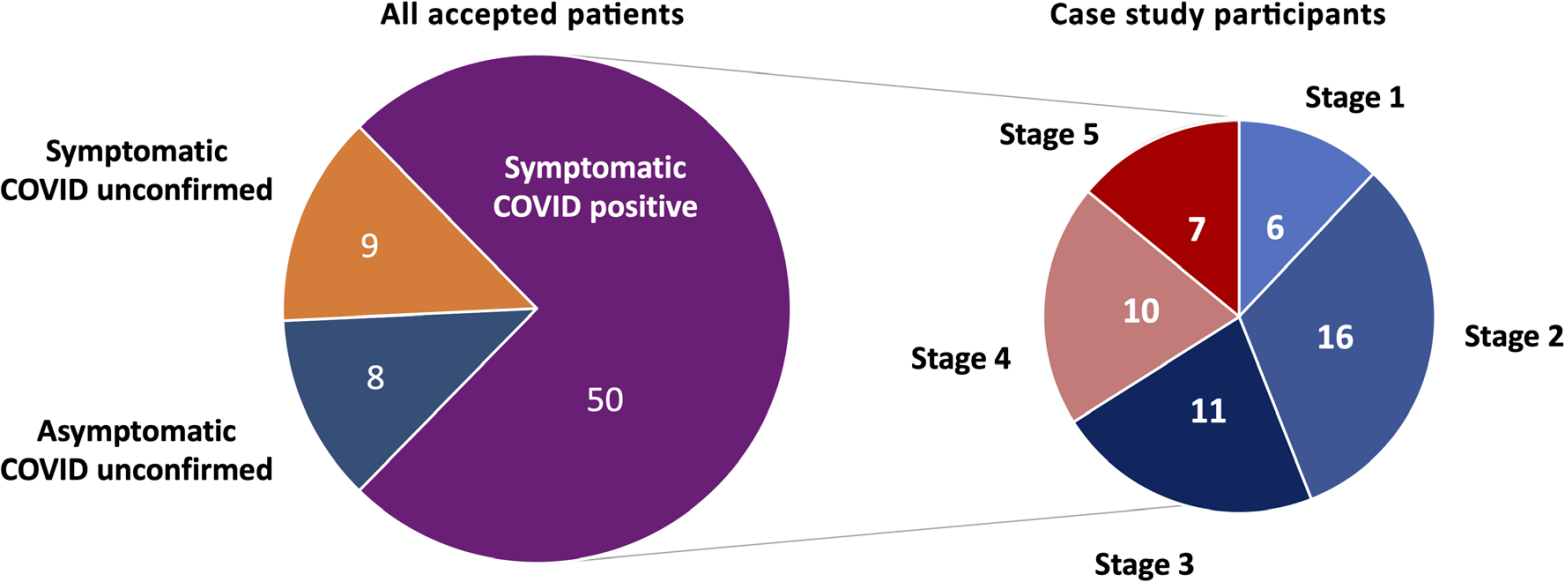
Patient and case study population statistics

Treatments were administered by physicians and/or under doctor supervision. Using ethical standards for compassionate care, all patients requesting therapy were treated free of charge and without regard to their severity, age or demographics. No patient was recruited or encouraged to receive PBM therapy, nor was any compensation or payment made to any patient receiving PBM therapy. Of their own volition (or after discussions with their personal physician), all candidates chose PBM as their primary therapeutic modality and elected not to be hospitalized. As such, no chest X‐rays were taken or available for the patients treated. Prior to therapy, COVID‐19‐positive patients were categorized in levels of increasing physiological distress, identified by five degrees of symptomatic severity, from mild to severe. Consistent with the observed pathology of COVID‐19 disease progression, the degrees of severity are herein referred to as “stages.” The nonrandomized sample population of all patients and the disease stage of identified case study members is represented in Figure 2.

Disease symptoms for COVID‐19 included malaise, dyspnea, cough, taste and smell loss, sinus inflammation, headaches (HA) and body aches (BA), abdominal discomfort (AbD) or cramping, fever and depressed SpO_2_ levels. A statistical analysis of symptoms in the incoming COVID‐19 patient population “n” is summarized in Table 1.

**TABLE 1:** Observed conditions for incoming COVID patient

Stage	n	Condition	COVID+	TTTx	Malaise	Dyspnea	Cough	T&S loss	Sinus inflammation	Aches & pain	Temperature (°F)	SpO_2_	HR
1	6	Mild	Y	2.5	2/6	0/6	2/6	1/6	**4/6**	HA	98.9	99	75
2	16	Mild to moderate	Y	2.0	**15/16**	1/16	**11/16**	1/16	**6/16**	HA	100.0	96	76
3	11	Moderate	Y	2.7	**11/11**	0/11	**9/11**	1/11	**7/11**	BA, HA, AbD	100.1	96	77
4	10	Moderate to severe	Y	4.0	**10/10**	2/10	**9/10**	2/10	**6/10**	BA, HA, AbD	100.4	93	82
5	7	Severe	Y	7.8	**7/7**	**6/7**	**6/7**	1/7	**5/7**	BA, HA, AbD	100.4	89	85
All	50	All	Y	—	45/50	9/50	37/50	6/50	28/50	BA, HA. AbD	—	—	—

*Note*: Bold number represent dominant symptoms in a given stage.

Abbreviations: AbD, abdominal discomfort; BA, body ache; HA, headache; HR, heart rate; T&S, taste and smell; TTTx, time‐to‐treatment.

General observations regarding the COVID‐19 patients include the following:

- 100% of all patients were confirmed by rapid test to be infected with COVID‐19.
- 50/50 patients were symptomatic, with 34% severe or moderately severe.
- 90% of all patients suffered malaise.
- 40/50 patients had fever, 26% with temperatures of ~101°F (38°C), the highest fever observed.
- 40/50 patients experienced BA and pains, and 100% of BA correlated with fever.
- 9/50 patients suffered shortness of breath or wheezing (dyspnea), mostly in severe stages.
- 37/50 patients had a cough, 30% of which was severe.
- 28/50 patients had rhinitis, sinus congestion and upper respiratory distress.
- 1/50 patients experienced severe eye inflammation.
- 2/50 patients had mild nausea, 3/50 reported abdominal distress and cramping (AbD)
- No patients in the study were on mechanical ventilators.
- Three patients supplemented room air with 6 L/min pure oxygen (100% FiO_2_).

Analysis of measured and reported symptoms in the table shows Stage 1 is characterized by sinus infection and a low‐grade fever but minimal coughing or lung dysfunction (as evidenced by a SpO_2_ of 99). For the case study patients, the data suggest initial COVID infection occurred in the sinuses and not in the lungs, bronchial passages or alveoli.

Stage 2, a mild‐to‐moderate degree of severity, is predominantly identifiable by a significant increase in coughing, the onset of 100°F (37.8°C) fever, and a 3‐point decline in SpO_2_ with a corresponding sense of worsening malaise. Although sinus inflammation persists, this stage appears to chronicle the migration of the infection from the sinuses into the lungs. In moderate Stage 3, a dry and hacking cough becomes pervasive while sinus inflammation and malaise continue. Persistent fever brings with it chills, BA, headaches and some abdominal cramping, but minimal reports of nausea. Stage 4, the onset of moderately severe acute symptoms sees another 3‐point decline in SpO_2_, a slightly higher degree of fever, widespread malaise, coughing and sinus congestion and the first reported incidences of dyspnea.

Stage 5, the severe acute disease phase, exhibits nearly universal symptoms of dyspnea, and coughing fits along with a precipitous drop in SpO_2_ into the 80s. In its mature infectious stage, COVID patients reportedly exhibit severe constriction of the bronchial passageways, lung congestion and possible organ damage (including the formation of scar tissue) consistent with dyspnea, low blood oxygen, coughing and chest pain. In one patient, even the smallest cough provoked painful paroxysmal 20‐minute coughing fits and an associated shortness of breath. In another patient, persistent sinus congestion further led to severe inflammation of the conjunctiva and mucus membranes surrounding both eyes but especially in their left eye. No subjects exceeding Stage 5 were studied as these patients were already hospitalized and not available for outpatient PBM treatments.

## 3 RESULTS

The following Table 2 represents the distribution of therapeutic results of PBM treatments as categorized and arranged by stages. The table contains the population of each group, confirmation of a COVID‐positive test for all patients in the study, the range (and average) time‐to‐treatment (TTTx; in days) for each group, the range (and average) number of PBM sessions performed on each group, the total time (TTAR; in days) for relief of acute symptoms and a description of the symptoms relieved, average of the total time (TTFR) for full recovery of the entire population of the group, and the corresponding range (and average) measured change in SpO_2_ for the group over the full course of the study.

**TABLE 2:** Summary of therapeutic results of PBM sessions

Stage	n	Condition	COVID+	TTTx	TTTx (average)	# of sessions	Sessions (average)	TTAR	Acute relief	TTFR	Final *Δ*SpO_2_	*Δ*SpO_2_ (average)
1	6	Mild	Y	1‐2	2.5	1	1.0	1.0	Sinuses, HA	2.0	0‐1	0.0
2	16	Mild to moderate	Y	1‐2	2.0	1‐2	1.1	0.9	Fever, cough, sinus, HA	1.6	0‐4	1.7
3	11	Moderate	Y	2‐6	2.7	1‐3	1.6	1.0	Fever, cough, sinus, BA/HA	3.5	0‐3	1.0
4	10	Moderate to severe	Y	3‐6	4.0	1‐3	2.1	1.0	Fever, cough, sinus, BA/HA	4.0	2‐6	4.0
5	7	Severe	Y	6‐14	7.8	1‐9	3.0	1.3	Dyspnea, fever, cough, sinus	6.0	3‐15	6.6

Abbreviations: BA, Body ache; HA, headache; TTAR, time‐to‐acute‐relief; TTTx, time‐to‐treatment; TTFR, time‐to‐full‐recovery.

**FIGURE 3:**
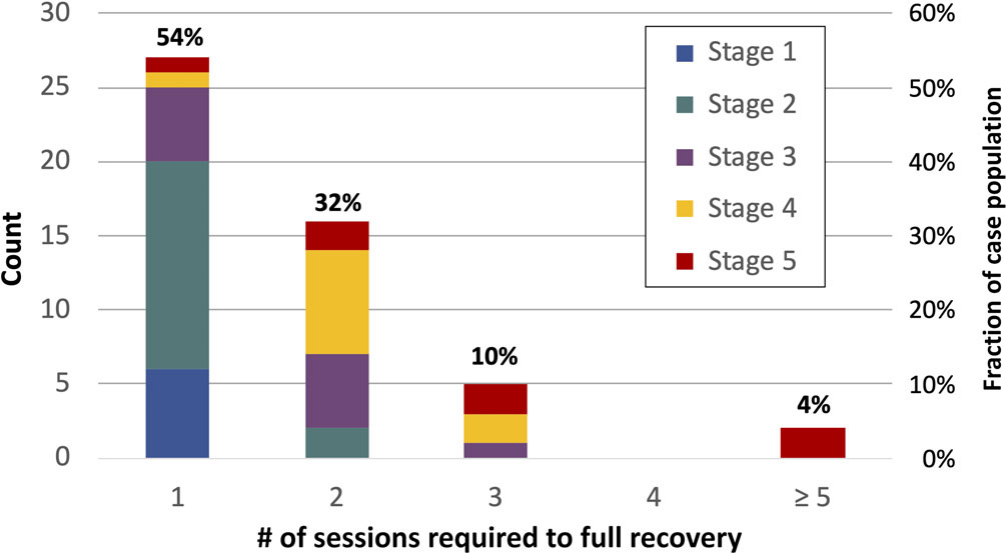
Histogram of number of photobiomodulation sessions performed to patient full recovery

As shown in Figure 3, a histogram of the number of treatments performed for the COVID‐positive case study group was limited to a maximum of three sessions for Stage 1 to Stage 4 patients. In contrast, two of seven Stage 5 patients required a greater number of PBM sessions (specifically seven and nine sessions) to restore normal breathing and sustained blood oxygen levels. Out of a preponderance of caution, one of these patients suffering from severe lung dysfunction concurrently received a 6‐day steroidal course as an anti‐inflammatory supplement to their PBM regimen.

**FIGURE 4:**
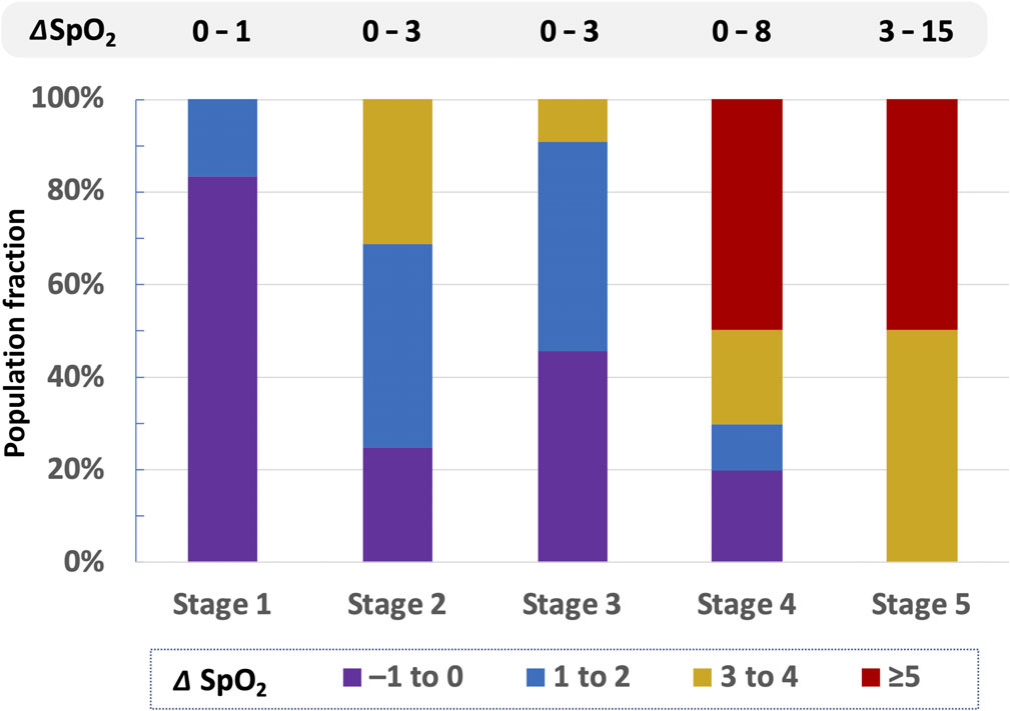
Observed increase in SpO_2_ from photobiomodulation regimen for progressive stages of COVID‐19

The data reinforces the general premise that left unchecked, COVID‐19 is selective, causing severe lung damage and dyspnea in a subset, but not all, of the infected population—especially to those sensitive to severe inflammation with a propensity for cytokine storms and autoimmune response. Another measure of lung dysfunction is evidenced by observed improvement in SpO_2_ levels during PBM and their correlation to the various stages of disease progression as shown in Figure 4. For patients suffering severe dyspnea and low oxygen saturation, PBM is shown to increase SpO_2_ by as much as 15 points, from 84 to 99.

**FIGURE 5:**
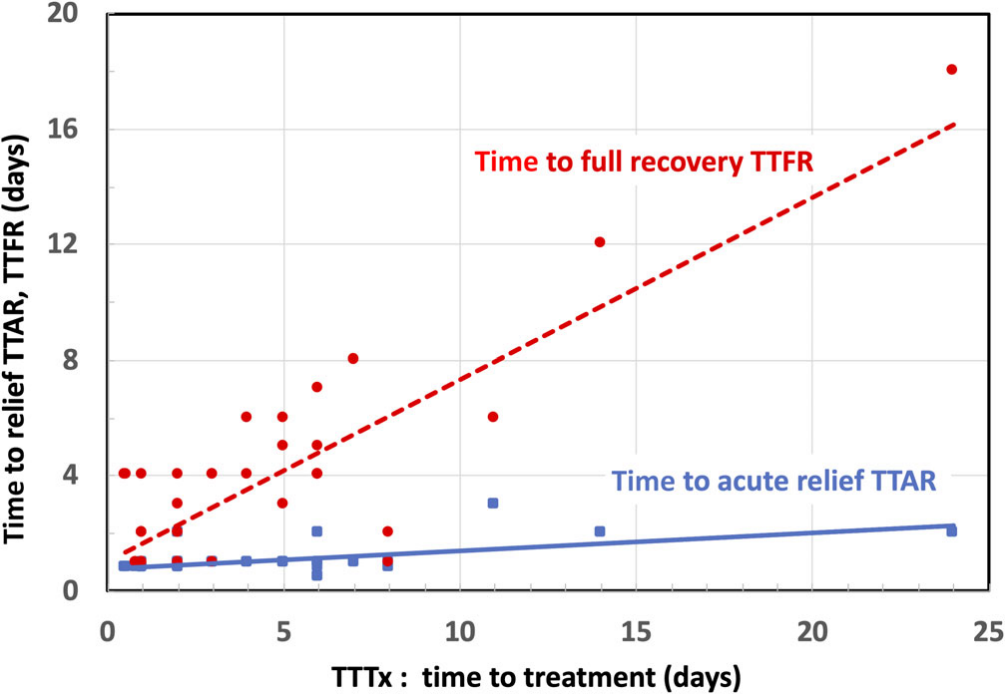
Scatter plot of time‐to‐acute‐relief (TTAR) and time‐to‐full‐recovery (TTFR) vs. time‐to‐treatment (TTTx)

In general, the rapidity by which a patient is able to fully recover from symptomatic COVID‐19 infections depends on how long they are ill before taking action, that is, TTFR monotonically correlates with the TTTx. This correlation is shown by the dashed line and circle markers in the scatter plot of Figure 5 which illustrates treatments performed within a week of the first symptoms of COVID‐19 can be resolved within 8 days and most within 4 days. By delaying treatment, full recovery of unventilated patients can take nearly 3 weeks to substantially resolve.

**FIGURE 6:**
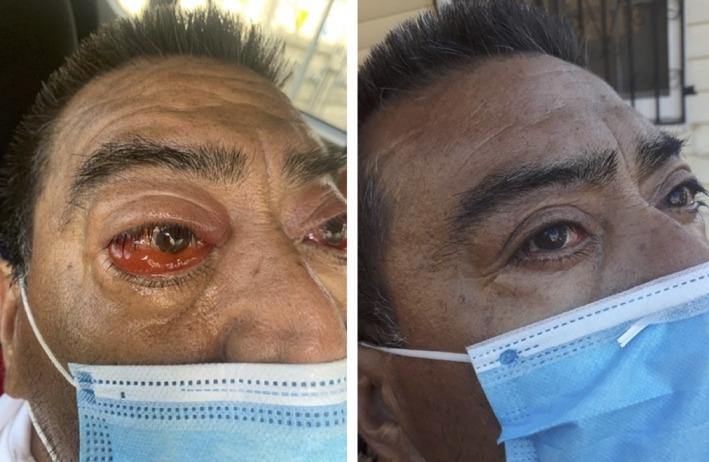
Reduction of eye inflammation after two photobiomodulation sessions

Curiously, the time required to substantially ameliorate acute COVID‐19 symptoms (shown by the solid line and square markers) occurs quickly, in approximately 3 to 4 days irrespective of the TTTx time lag before commencing treatment. Acute condition relief of COVID‐19 includes breaking fever, improving breathing, reducing painful dry coughing, eliminating sinus congestion and relieving severe eye inflammation. For example, a Stage 5 COVID‐19 patient suffering severe eye inflammation showed significant reduction in inflammation of the conjunctiva and mucous membranes of the eye after two PBM sessions in a 4‐day interval, as shown in Figure 6.

Another aspect of dynamic PBM used in this study is the application of sequential algorithmic variable‐frequency treatment protocols. As described in Section 2.3 of this paper, each treatment phase performs a different role therapeutically. Consistent with published literature, this study reinforces the argument for frequency‐dependent tissue specificity. For example, one patient suffering from severe dry cough and dyspnea experienced coughing fits continuing unabated for 20 minutes causing significant breathing distress. Although during PBM the coughing continued throughout the first phase of the therapy intended to systemically enhance immune response, immediately after commencing the phase for enhancing circulation, the patient's coughing suddenly decreased by 95%, anecdotally reinforcing the premise that in dynamic PBM, different modulation frequencies enhance tissue specificity. Further studies on the role of pulse frequencies on PBM modalities are warranted.

## 4 DISCUSSION

The profoundly different time scales for TTAR and TTFR and their distinct dependences on TTTx suggest that different biophysical mechanisms are involved in the acute and long‐term recovery phases for COVID‐19 using therapeutic PBM. Specifically, the nearly immediate relief of acute conditions, irrespective of the time a patient was COVID‐19 symptomatic before commencing therapy, suggests activation of photochemical processes not related to NTFs or gene expression. Mechanistically, the absorption of photons by the mitochondrial chromophore and transmembrane protein CCO is known to rapidly release NO resulting in vasodilation, increased tissue and blood oxygen levels and enhanced circulation.

This effect, combined with a propensity for erythrocytes to become negatively charged during PBM and thereby resisting clumping (through repelling electrostatic force), enhances blood perfusion and reduces the chance of thrombosis, edema and hypoxemia‐induced tissue necrosis in the bronchia and alveoli. By suppressing local inflammation, enhanced circulation also helps to avoid an overreaction of the immune system precipitating a dangerous cytokine storm.

Concurrently, the release of superoxides, H_2_O_2_ and other ROS during PBM initiates an immediate innate immune response that is able to directly attack invading viral pathogens such as coronavirus at the molecular level while preventing the onset of secondary bacterial infections. The innate immune response is further assisted kinetically by a rapid increase in cellular metabolism resulting from a higher MMP during PBM and a consequential rate increase in ATP production, peaking 6 hours after a treatment.

In contrast, full recovery from a COVID‐19 infection requires repair of the damage to the lungs, mucus membranes and other epithelial tissue caused by the viral infection starting by removing dying or damaged cells and scar tissue. Effecting tissue repair involves nuclear transcription to produce enzymes, protein, and catalysts needed to expedite the healing process including inflammation, regeneration and remodeling of damaged tissue and to selectively perform cellular necrosis, phagocytosis and fibroblastic regrowth. The cumulative magnitude of damage to be repaired is therefore proportional to the time a patient was infected and symptomatic before commencing PBM. And since a greater degree of tissue repair takes longer, it is not surprising the TTFR would be monotonically proportional to the TTTx while TTAR is not.

## 5 CONCLUSIONS

A therapeutic PBM protocol comprising a single 84‐minute concurrent treatment of the sinuses and anterior lungs using whole‐organ dynamic PBM has demonstrated significant promise in the symptomatic treatment of COVID‐19. Performing PBM on a 50‐person nonrandomized sample of symptomatic unventilated COVID‐19 patients using PBM resulted in all 50 patients fully recovering symptom free within 3 weeks, with the majority of the sample population recovering within 4 days. The PBM therapeutic modality offers numerous advantages over pharmacological regimens and exhibits no adverse side effects on the kidneys, liver or stomach.

Although early treatment was found to reduce the severity of the disease and shorten the TTFR, the time required to deliver immediate symptomatic relief of acute conditions was less than 2 to 3 days and generally required only a single PBM session irrespective of the time a patient was COVID‐19 symptomatic before commencing therapy. Acute symptoms ameliorated by PBM include improving breathing, reducing painful dry coughing, breaking fever, eliminating sinus congestion, and relieving severe eye inflammation. The time required to fully recover from all residual effects of a COVID‐19 infection, however, was longer—taking up to 3 weeks in duration roughly proportional to the interval when a patient first became COVID symptomatic until the time when PBM therapy commenced. The use of whole‐organ dynamic PBM delivered via 3D‐conforming LED pads shows promise as a therapeutic regimen in the non‐pharmacological treatment of COVID‐19.

Given the significant recovery (50/50) of COVID‐positive patients to PBM therapy demonstrated in this study, the use of whole‐organ dynamic PBM is favorably indicated for the symptomatic relief of COVID‐19.

Although further research is needed to optimize treatment protocols (and ideally shorten session times to 1 hour), an emergency use authorization for this treatment to combat the ongoing worldwide COVID‐19 pandemic is warranted. Moreover, the widespread application of the PBM protocols described herein delivered through outpatient therapy, clinics or in acute care centers can relieve hospital overcrowding during pandemic surges, both by reducing the number of severely ill patients and by shortening recovery from severe symptoms to under 4 days.

## CONFLICTS OF INTEREST

No doctors involved in this study are shareholders, employees or paid consultants of Applied BioPhotonics Ltd. (ABP), the device manufacturer, system specifier and IP holder. No doctors received compensation from ABP to participate in this study. No patients were paid for receiving PBM therapy in this study. No patients requesting therapy were turned away from receiving treatments. LightMD Inc. is the US FDA‐registered, licensed importer and distributor of ABP products in the United States.

## Data Availability

Data sharing is not applicable to this article as no new data were created or analyzed in this study.
